# Is Performance of Fluorine-18-fluorodeoxyglucose Positron Emission Tomography/Computed tomography (CT) or Contrast-enhanced CT Efficient Enough to Guide the Hilar Lymph Node Staging for Patients with Esophageal Squamous Cell Carcinoma?

**DOI:** 10.3389/fonc.2022.814238

**Published:** 2022-02-25

**Authors:** Li Chu, Shuai Liu, Tiantian Guo, Liqing Zou, Bin Li, Jianjiao Ni, Xi Yang, Xiao Chu, Fei Liang, Yida Li, Yuyun Sun, Qiao Li, Fang Yin, Guodong Li, Zhengfei Zhu

**Affiliations:** ^1^ Department of Radiation Oncology, Fudan University Shanghai Cancer Center, Shanghai, China; ^2^ Institute of Thoracic Oncology, Fudan University, Shanghai, China; ^3^ Department of Oncology, Shanghai Medical College, Fudan University, Shanghai, China; ^4^ Shanghai Key Laboratory of Radiation Oncology, Shanghai, China; ^5^ Department of Nuclear Medicine, Fudan University Shanghai Cancer Center, Shanghai, China; ^6^ Department of Thoracic Surgery, Fudan University Shanghai Cancer Center, Shanghai, China; ^7^ Department of Biostatistics, Zhongshan Hospital, Fudan University, Shanghai, China; ^8^ Department of Radiology, Fudan University Shanghai Cancer Center, Shanghai, China; ^9^ Center for Drug Clinical Research, Shanghai University of Traditional Chinese Medicine, Shanghai, China; ^10^ Department of Interventional Radiology, Fudan University Shanghai Cancer Center, Shanghai, China

**Keywords:** esophageal squamous cell carcinoma, PET, CT, hilar lymph node, radiotherapy

## Abstract

**Introduction:**

We evaluated the diagnostic performance of fluorine-18-fluorodeoxyglucose (18F-FDG) positron emission tomography (PET)/computed tomography (CT) and contrast-enhanced CT in the detection of hilar lymph node metastasis (LNM) in esophageal squamous cell carcinoma (ESCC) to determine their value in guiding hilar lymph node staging and delineating radiation target volume.

**Methods:**

Consecutive patients with ESCC who underwent both PET/CT and contrast-enhanced CT before radical lymphadenectomy and esophagectomy at our institution from September 2009 to November 2018 were enrolled. The sensitivity (SE), specificity (SP), positive predictive value (PPV), and negative predictive value (NPV) of FDG-PET/CT and contrast-enhanced CT for diagnosing hilar LNM were calculated.

**Results:**

Of the 174 patients included, contrast-enhanced CT predicted nine positive cases, while PET/CT predicted one, and eight (4.6%) were identified as pathologically positive for their resected hilar lymph nodes. The SE, SP, PPV, and NPV of PET/CT and contrast-enhanced CT were 0.000, 0.994, 0.000, and 0.954; and 0.125, 0.952, 0.111, and 0.958, respectively. The specificity showed a significant difference (P=0.037). PET/CT is slightly more specific than contrast-enhanced CT.

**Conclusions:**

PET/CT and contrast-enhanced CT may be useful tools for predicting the negativity of hilar LN status, but they are not recommended for guiding the hilar lymph node staging and the delineating of hilar LNM in radiotherapy planning of ESCC patients based on their low PPV.

## Introduction

Esophageal cancer is one of the leading causes of death from cancer (1). More than 90% of cases with esophageal carcinoma in China are esophageal squamous cell carcinoma (ESCC) (2). ESCC is a tumor type prone to lymph node metastasis (LNM), which is one of the most vital prognostic factors in ESCC patients (3, 4). Based on the operative pathology, LNM has been found to be involved in more than half of surgical patients in large-scale retrospective analyses (5, 6). However, among LN of ESCC, the incidence of hilar LNM is relatively low. In a retrospective analysis involving 1361 patients with thoracic ESCC who underwent curative esophagectomy, 52.5% (714/1361) were found to have LNM, while only 1% and 2.5% of patients experienced left and right hilar LNM, respectively (6).

Radiotherapy, which targets the primary tumor and involved LNs, has a well-established role in the management of ESCC. To delineate the target volume, reliable imaging techniques for detecting involved lymph nodes are of critical importance to ensure accurate coverage of the disease, which is also a determining factor of selecting patients with curative treatment. It is not uncommon for imaging examination to report hilar LN abnormalities in patients with ESCC in routine clinical practice. Thus, the assessment of hilar lymph node involvement in ESCC is clinically relevant, as the inclusion of hilar lymph node into the target volume can increase radiation doses to surrounding normal structures, particularly to the lungs, thereby potentially increasing the risk of normal tissue complications.

Fluorine-18-fluorodeoxyglucose Positron Emission Tomography (FDG-PET)/CT and contrast-enhanced CT are among the most common imaging modalities to evaluate the status of hilar lymph node, Unfortunately, the number of studies on their accuracy for diagnosing hilar LNM is very limited and it is very difficult to draw a solid conclusion. A recent investigation demonstrated that none of the biopsied PET/CT-positive hilar nodes (n=4) confirmed the presence of metastases, which has raised concern over the diagnostic performance of (PET)CT in the detection of hilar LNM (7). To provide further insight into this issue, we performed a retrospective study of a relatively larger cohort in patients with ESCC who underwent both FDG-PET/CT and contrast-enhanced CT before radical lymphadenectomy and esophagectomy, focusing on the diagnostic performance of FDG-PET/CT and contrast-enhanced CT for hilar lymph node.

## Materials and Methods

### Patients

**Figure 1:**
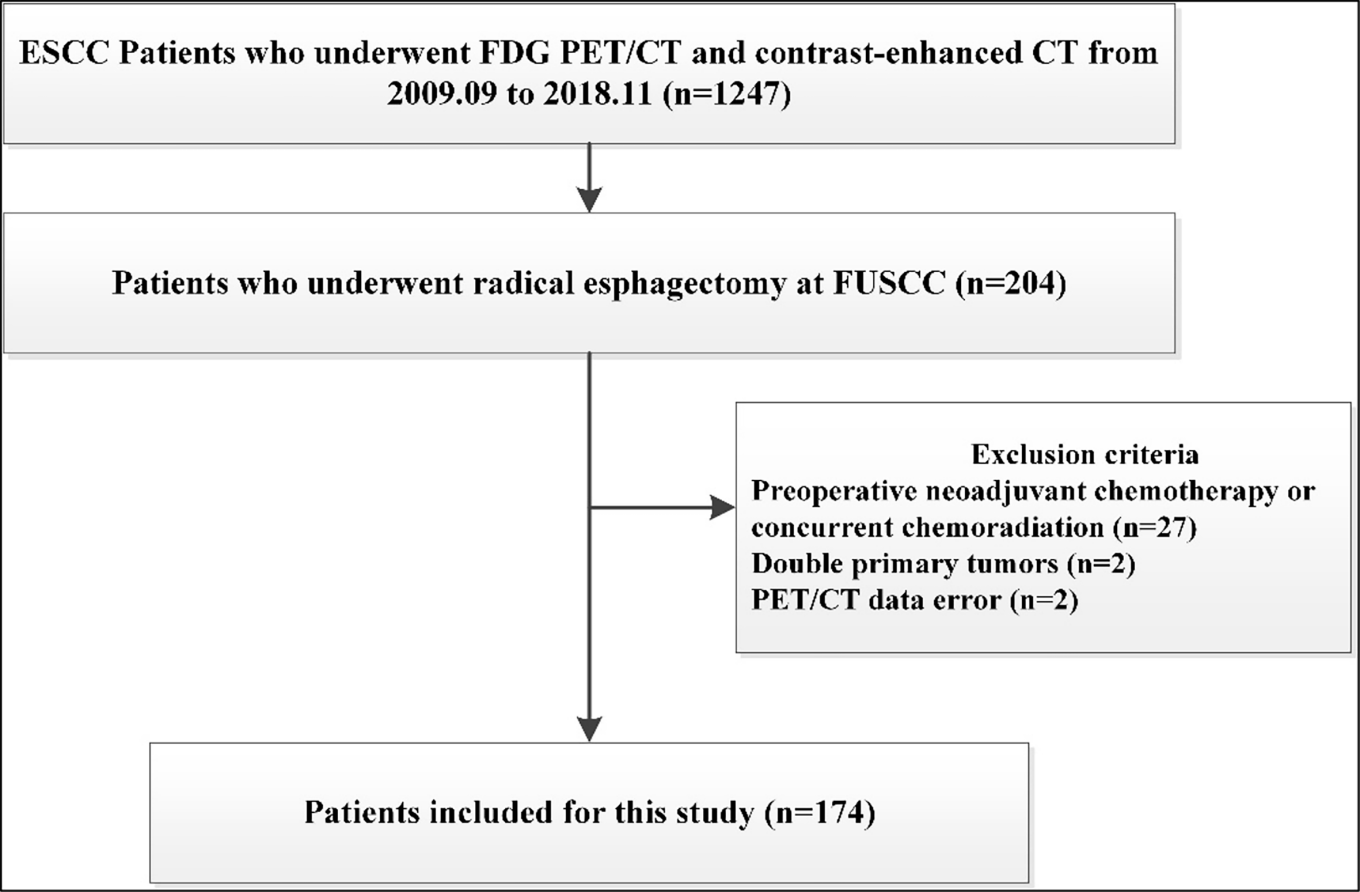
Patient disposition chart.

The database of ESCC was approved by the institutional review board of our Cancer Center for this study. From September 2009 to November 2018, a total of 1247 patients with ESCC received FDG-PET/CT and contrast-enhanced CT at our Cancer Center. Among them, 212 patients undergoing radical lymphadenectomy and esophagectomy were retrospectively analyzed. We excluded patients who underwent preoperative chemotherapy or concurrent chemoradiation before surgery (n=27), because these treatment modalities may interfere with nodal status. Patients who underwent non-radical esophageal cancer surgery and not having histopathological examination of hilar lymph node were also excluded (n=8). Additionally, there were two patients with double primary tumors, and two patients for whom there were data errors of their FDG PET/CT scans. Thus, a total of 174 patients were further evaluated and included in the study (**Figure 1**).

### FDG PET/CT Procedure and Evaluation

18F-FDG was produced by cyclotron using the Explora FDG4 module at Fudan University Shanghai Cancer Center. The radiochemical purity was over 95%. All patients fasted at least 6 hours before imaging. After injecting 7.4 MBq/kg (0.2 mCi/kg) 18F-FDG, patients were kept relaxed for approximately 1 hour. Images were obtained on a Siemens biograph 16HR PET/CT scanner (Knoxville, Tennessee, USA).

The images were reviewed and manipulated in a multimodality computer platform (Syngo, Siemens, Knoxville, Tennessee, USA). Standardized uptake value (SUV) of lymph nodes = [decay-corrected activity (kBq)/tissue volume (ml)]/[injected 18F-FDG activity (kBq)/body mass (g)]. All PET/CT images were analyzed by two senior nuclear medicine physicians independently according to clinical index and image performance, and did so together for the retrospective study in which they were blinded to the pathological results. Taking the liver as a reference organ, nodes were diagnosed as “involved” *via* PET/CT if the nodes were implicated *via* CT and the relevant component exhibited FDG uptake that was greater than background.

### Contrast-Enhanced CT Procedure and Evaluation

All patients underwent scanning on a Somatom Definition AS scanner (Siemens Healthcare, Erlangen, Germany). Breath-hold training was performed before each examination. All patients were asked to hold their breath at the end of inspiration as long as possible. All injections were performed with an automatic power injector with which 90 ml of contrast medium (Optiray 350 mgI/ml; Mallinckrodt Medical, St. Louis, MO, USA) was injected into the antecubital vein at a rate of 4 ml/s. Contrast-enhanced images were acquired at 90 s after injection. Imaging was performed from the thoracic inlet to the middle portion of the kidneys. Scanning parameters were as follows: 120 kVp, dose modulation ACS (Brilliance-iCT), or 50–100 mA (GE HD750 and Somatom Definition AS), slice thickness 1 mm; matrix 512×512 and standard resolution algorithms.

Various CT scanning criteria have been used to define malignant involvement of lymph nodes, and there is no node size that can reliably determine the stage. In the present study, a short-axis lymph node diameter of ≥1 cm on a CT scan was chosen as the criterion for malignancy due to its wide use in clinical practice (8, 9).

### Surgery and Pathology

Transthoracic esophagectomy and extensive lymph node dissection were performed by experienced thoracic surgeons. Of note, all patients received total mediastinal lymphadenectomy that included bilateral hilar lymph node dissection, and each dissected lymph node group was labelled according to a modified lymph node mapping system for esophageal cancer (10).

### Statistical Analysis

Statistical Package for the Social Sciences version 25.0 (IBM Corp., Armonk, NY, USA) was used to performed statistical analysis. The sensitivity, specificity, positive predictive value, negative predictive value, and accuracy of FDG-PET/CT and contrast-enhanced CT for the assessment of hilar LNM were determined using pathological results as reference standards. Continuous data were collected as means and standard deviations, or medians and range. Classification data were collected as numbers and percentages. The Chi-square test or Fisher exact test was used to compare categorical data. Two-sided P values<0.05 were considered statistically significant.

## Results

### Baseline Characteristics

The baseline characteristics are shown in **Table 1** according to the 7th edition of the American Joint Committee on Cancer staging. Of the 174 patients, 144 (82.8%) were men, and 30 (17.2%) were women. The median age was 63 (range: 45–79) years. The median interval between PET/CT examinations and surgery was seven days (range 1–65 days), and 10 days (range 1–65 days) between contrast-enhanced CT and surgery. A total of 5749 lymph nodes were dissected, including 210 hilar lymph nodes. The metastatic mediastinal lymph nodes were distributed in the regions of 106 (33.0%), 110 (20%), 108 (15.7%), 109 (13.0%), 107 (7.8%), 105 (7.0%), and 112 (3.5%). The median SUVmax for hilar lymph nodes was 0 (range: 0–9.7). The median size of the short diameter of the hilar lymph nodes examined under contrast-enhanced CT was 0.6 (range: 0–1.3) cm. Only eight (4.6%) patients were identified as positive for their resected hilar lymph nodes by pathological examination, demonstrating that hilar lymph node metastasis is a rare event in ESCC patients. As shown in **Table 2**, no clinicopathological factors could predict pathological hilar LNM.

**Table 1:** Patient and tumor characteristics.

Characteristics	N = 174 (%)
**Age (years)**	
Median (range)	63 (45-79)
≥60	118 (67.8)
<60	56 (32.2)
**Gender**	
Male	144 (82.8)
Female	30 (17.2)
**Alcohol intake**	
Never	83 (47.7)
Ever	91 (52.3)
**Smoking history**	
Never	51 (29.3)
Ever	123 (70.7)
**Tumor location**	
Upper	11 (6.3)
Middle	111 (63.8)
Lower	52 (29.9)
**Pathologic T category**	
T1-2	83 (47.7)
T3-4	91 (52.3)
**Pathologic N category**	
N0-1	136 (78.2)
N2-3	38 (21.8)
Number of lymph node dissections^*^	32 (7-85)
**Tumor, SUV max** ^*^	10.2 (0-33.2)
**Hilar LN, SUV max** ^*^	0 (0-9.7)
**Hilar LN, size (measured by CT, cm** ^*^	0.6 (0-1.3)

^*^Date shown as median (range).

**Table 2:** Factors associated with hilar lymph node metastasis.

Characteristic	Patients (n)		*P*
	Hilar LNs (+)	Hilar LNs (-)	
**Age**			0.272^1^
<60	4	52	
≥60	4	114	
**Gender**			0.354^1^
Male	8	136	
Female	0	30	
**Alcohol intake**			0.723^1^
Ever	5	86	
Never	3	80	
**Smoking history**			0.440^1^
Ever	7	116	
Never	1	50	
**Tumor location**			0.507^1^
Upper	1	10	
Middle	7	104	
Lower	0	52	
**Pathologic T category**			0.282^1^
T1-2	2	81	
T3-4	6	85	
**Hilar LN, size (measured by CT, cm)**			1.000^1^
<1.0	6	140	
≥1.0	2	26	
Tumor, SUV max^*^	11.4 (6.7-15.2)	10.2 (0-33.2)	0.656^2^

^*^Date shown as median (range).

^1^Measured by Fisher’s exact test.

^2^Measured by Student’s t test.

### Diagnostic Performance of PET/CT and Contrast-Enhanced CT in Hilar Lymph Node

Positive lymph nodes as determined *via* PET/CT were detected in only one patient (0.06%), but this patient did not exhibit pathological hilar LNM. Contrast-enhanced CT examination revealed nine positive cases; however, only 1 case was consistent with pathological examination. Detailed numbers of positive or negative cases of PET/CT and contrast-enhanced CT in diagnosing hilar lymph node are shown in **Table 3**.

**Table 3:** Number of positive cases of PET/CT and contrast-enhanced CT in diagnosing hilar lymph node (n = 174).

		Pathology	
		Positive	Negative
**PET/CT**	positive	0	1
	negative	8	165
**CT**	positive	1	8
	negative	7	158

As shown in **Table 4**, for hilar lymph node metastasis, both PET/CT and CT exhibited high specificity (99.4% and 95.2%) and negative predictive value (94.8% and 95.8%), but low sensitivity (0% and 12.5%) and positive predictive value (0% and 11.1%), suggesting that both are of limited value for this purpose. The specificity showed a significant difference (P=0.037). PET/CT is slightly more specific than contrast-enhanced CT.

**Table 4:** Diagnostic performance of PET/CT and contrast-enhanced CT in diagnosing hilar lymph node (n = 174).

	PET/CT n (95% CI)	CT n (95% CI)	*P* value^1^
**Sensitivity**	0.000 (0.000-0.402)	0.125 (0.007-0.533)	1.000^1^
**Specificity**	0.994 (0.962-1.000)	0.952 (0.904-0.977)	0.037^1^
**Positive predictive value**	0.000 (0.000-0.945)	0.111 (0.006-0.493)	1.000^1^
**Negative predictive value**	0.954 (0.908-0.978)	0.958 (0.911-0.981)	0.865^2^
**Accuracy**	0.948 (0.915-0.982)	0.914 (0.872-0.956)	0.290^2^

^1^Measured by Fisher’s exact test.

^2^Measured by Chi-square test.

## Discussion

Herein, we conducted a retrospective study with a relatively larger patient cohort to explore the diagnostic performance of FDG-PET/CT and contrast-enhanced CT for hilar LNM in patients with ESCC. Our results demonstrate that hilar LNM is a rare event in ESCC patients and both PET/CT and contrast-enhanced CT are of limited value for diagnosis and delineation of hilar lymph nodes in radiotherapy. The diagnostic value of PET-CT and enhanced CT in LNM of esophageal cancer has been explored by several studies (7, 11–13). The results from previous studies revealed low specificity of PET-CT or enhanced CT for detection of LNM. It is noteworthy that the eighth edition AJCC cancer staging does not consider hilar lymph nodes as regional nodes for esophageal cancer. Unique features and strengths of our study compared to previous investigations included the relatively larger patient cohort and the unique insights it offers into the clinical value of PET/CT and contrast-enhanced CT for accurate staging according to the eighth edition AJCC cancer staging system, focusing particularly on the diagnostic performance of (PET)CT and enhanced CT in the detection of hilar LNM. In our study, both PET/CT and contrast-enhanced CT exhibited high specificity (SP) and negative predictive value (NPV), but low sensitivity (SE) and positive predictive value (PPV), which is consistent with previous studies conducted for NSCLC (9, 14–16). There may be several reasons for this phenomenon. Lymph node enlargement can be caused by tumor metastasis, reactive hyperplasia, or granulomatous inflammation, and high FDG uptake is often caused by sarcoidosis, sarcoid-like reactions, or an infection. These will all cause trouble in the specificity of differential diagnosis (17). Due to the limited resolution, scatter effects, and attendant motion artifacts caused by esophageal and stomach peristalsis, PET/CT may also not be sensitive enough to detect metastases in lymph nodes.

Currently, radiation therapy is well recognized as an important part of treatment for esophageal cancer (18, 19). It is noteworthy that, given the anatomical characteristics of hilar LNM in ESCC, effective imaging of hilar LNM is critically important to guide radiotherapy treatment planning for these patients. However, based on our current research, the delineation of target volume using PET/CT or contrast-enhanced CT may lead to an unnecessarily aggressive treatment in a number of ESCC patients due to the limited positive predictive value for hilar LNM. Therefore, pathologic examination of suspicious hilar LNM by PET/CT or contrast-enhanced CT is encouraged.

There is a growing appreciation for the role of EBUS-TBNA in detecting hilar LNM. EBUS-TBNA is regarded as a safe and minimally invasive technique for sampling hilar lymph node, with an NPV of 91–99% and PPV of 92.4–100% (20–23). The utility of EBUS-TBNA for the evaluation of suspicious hilar LNM in ESCC has also been assessed by Schurink et al. They found 2.5% (21/857) patients had the positive hilar LNM at staging (11 ESCC, 10 Adenocarcinoma). Of those, 4 had successful biopsies (EBUS, CT-guided fine needle aspiration or tru-cut biopsy) and none were positive, and no recurrence of disease was seen during follow-up in these patients (7). indicating the false positive PET/CT results. However, the utility of EBUS-TBNA for the evaluation of suspicious hilar LNM in ESCC needs further investigation.

The most important limitation of this study is that there were only 8 patients in the hilar lymph node metastasis group. Thus, the diagnostic sensitivity, specificity, and positive predictive value in the CT group and PER/CT group were compared by use of Fisher’s exact test. In summary, PET/CT and contrast-enhanced CT may be useful tools for predicting the negativity of hilar LN status, but they are not recommended for delineation of hilar LNM in the radiotherapy planning of ESCC patients due to their low PPV. All cases included in this study received radical operations, representing a population with relatively early-stage disease, and this may limit the generalizability. However, we found that the T stage could not predict the incidence of hilar LNM, and previous studies have shown that hilar LN recurrences are relatively low after definitive chemoradiotherapy for locally advanced ESCC (24–26). However, we think further studies regarding pathological diagnosis using minimally invasive techniques for suspected hilar LNs are warranted.

## Data Availability Statement

The raw data supporting the conclusions of this article will be made available by the authors, without undue reservation.

## Ethics Statement

The studies involving human participants were reviewed and approved by the institutional review board of Fudan University Shanghai Cancer Center. Written informed consent for participation was not required for this study in accordance with the national legislation and the institutional requirements.

## Author Contributions

ZZ and GL designed the research. LC, SL, and TG interpreted and discussed the results. LC, SL, TG, LZ, BL, JN, XY, XC, FL, YL, YS, QL, and FY collected the data, analysed the data, wrote the manuscript. All authors read and approved the final manuscript.

## Funding

The research was supported by National Natural Science Foundation of China (82003230, 81703024).

## Conflict of Interest

The authors declare that the research was conducted in the absence of any commercial or financial relationships that could be construed as a potential conflict of interest.

## Publisher’s Note

All claims expressed in this article are solely those of the authors and do not necessarily represent those of their affiliated organizations, or those of the publisher, the editors and the reviewers. Any product that may be evaluated in this article, or claim that may be made by its manufacturer, is not guaranteed or endorsed by the publisher.
